# The Role of Thyroid Hormone Signaling in the Prevention of Digestive System Cancers

**DOI:** 10.3390/ijms140816240

**Published:** 2013-08-06

**Authors:** Adam R. Brown, Rosalia C. M. Simmen, Frank A. Simmen

**Affiliations:** 1 Interdisciplinary Biomedical Sciences Program, University of Arkansas for Medical Sciences, Little Rock, AR 72205, USA; E-Mails: brownadamr@uams.edu (A.R.B.); simmenrosalia@uams.edu (R.C.M.S.); 2 Department of Physiology and Biophysics, University of Arkansas for Medical Sciences, Little Rock, AR 72205, USA; 3 Department of Pediatrics, University of Arkansas for Medical Sciences, Little Rock, AR 72205, USA

**Keywords:** thyroid hormone, thyroid hormone receptor, esophageal cancer, gastric cancer, liver cancer, pancreatic cancer, colorectal cancer

## Abstract

Thyroid hormones play a critical role in the growth and development of the alimentary tract in vertebrates. Their effects are mediated by nuclear receptors as well as the cell surface receptor integrin α_V_β_3_. Systemic thyroid hormone levels are controlled via activation and deactivation by iodothyronine deiodinases in the liver and other tissues. Given that thyroid hormone signaling has been characterized as a major effector of digestive system growth and homeostasis, numerous investigations have examined its role in the occurrence and progression of cancers in various tissues of this organ system. The present review summarizes current findings regarding the effects of thyroid hormone signaling on cancers of the esophagus, stomach, liver, pancreas, and colon. Particular attention is given to the roles of different thyroid hormone receptor isoforms, the novel integrin α_V_β_3_ receptor, and thyroid hormone-related nutrients as possible protective agents and therapeutic targets. Future investigations geared towards a better understanding of thyroid hormone signaling in digestive system cancers may provide preventive or therapeutic strategies to diminish risk, improve outcome and avert recurrence in afflicted individuals.

## 1. Introduction

Follicular cells of the thyroid gland manufacture and secrete the thyroid hormones (THs) l-thyroxine, (T_4_) and l-triiodothyronine (T_3_). These hormones control the release of both thyrotropin-releasing hormone (TRH) from the hypothalamus and thyroid-stimulating hormone (TSH) from the anterior pituitary gland via negative feedback loops [1]. Of the two THs, T_4_ is produced more abundantly. Upon entering the bloodstream, T_4_ is transported to other organs in the body where it undergoes deiodination to the active thyroid hormone T_3_ by the selenoenzymes iodothyronine deiodinase I and II (D1 and D2) [2]. On the other hand, iodothyronine deiodoinase III (D3) serves to protect cells from excessive thyroid hormone signaling by inactivating T_3_ and preventing activation of T_4_ [3]. The liver and kidneys are the primary sites of conversion of T_4_ to T_3_, although a number of other tissues, including some gastrointestinal ones, are also capable of deiodination [3–5].

Thyroid hormones bind to thyroid hormone receptors (TRs), which are ligand-regulated transcription factors belonging to the nuclear receptor superfamily [6,7]. These receptors contain two zinc finger binding domains that are highly conserved across species [8]. Such domains bind to thyroid hormone response elements (TREs) in the promoter and/or enhancer regions of target genes [9]. The most typical TRE is comprised of a tandem repeat of two AGGT(C/A) sequences separated by four base pairs (direct repeat 4, DR4), although TREs can exist as palindromes or inverted palindromes. TREs can vary by the number of base pairs separating the tandem sequences [7,10,11]. Two thyroid hormone receptor genes, *THRA* and *THRB*, have been identified on human chromosomes 17 and 3, respectively [12,13]. Variable splicing of primary mRNAs gives rise to three different TRα protein isoforms (TRα1, TRα2, and TRα3) and two TRβ protein isoforms (TRβ1 and TRβ2) [14]. The TRs can bind to DNA as monomers, homodimers, or heterodimers with retinoid X receptor (RXR), the latter being the most transcriptionally active [9,14,15].

In addition to the intracellular TRs, the integrin α_V_β_3_ has more recently been identified as a cell surface receptor for TH [16]. Two separate binding sites for TH have been identified on the extracellular domain of this integrin. One site binds T_3_ to stimulate the PI3K pathway, leading to the translocation of TRα to the nucleus for transcription of HIF-1α; the other site binds primarily to T_4_ to activate ERK1/2 leading to nuclear localization of TRβ1 [17]. The latter mechanism has been demonstrated to enhance mitogenesis and decrease apoptosis in tumor cells [18,19]. T_4_ binding to this integrin has also been implicated in increased FGF-dependent angiogenesis [16,20]. Other studies have shown that tetraidothyroacetic acid (tetrac) can antagonize T_4_ binding to integrin α_V_β_3_, and thus inhibit tumor cell proliferation in tissue culture as well as in mouse xenograft models [18,19,21–24].

Thyroid hormone signaling plays a critical role in intestinal development and homeostatic control [25]. The fundamental role of TH in gut remodeling during amphibian metamorphosis has been well-documented [26–29]. These developmental changes are controlled by preprogrammed alterations in expression of the iodothyronine deiodinases that in turn regulate intracellular T_3_ concentration [30]. Other studies have found similar functions for TH in mouse intestinal development, although this is not the case for the human [31–33]. Thyroid hormone also controls physiological processes in the adult intestine, including the onset of intestinal alkaline phosphatase expression and absorption of fructose [34,35]. Further, TRα maintains intestinal crypt cell homeostasis by regulating the rate of cell renewal and apoptosis in response to DNA damage [36]. However, while some studies have characterized a role for TH in regulation of intestinal stem cells of amphibians, it is yet to be determined whether TH signaling plays a direct role in mammalian intestinal stem cell biology [37–39].

Given the important developmental and physiological functions of TH in the gut, a number of studies have examined the effects of TH on cancer prevention, development, and progression in various digestive tissues. Some of these relationships have been well characterized, while others remain poorly understood. This review focuses on the influence of TH signaling on multiple digestive system cancers with an emphasis on prevention and susceptibility.

## 2. Esophageal Cancer

Only a few studies have linked TH signaling to the development of esophageal cancer (EC). In one study, greater incidence of hyperthyroidism was demonstrated in patients with EC compared to controls [40]. By contrast, a recent study found that expression of TRβ1 mRNA was significantly reduced in esophageal cancers compared to normal esophageal mucosa [41]. In agreement with the latter, loss of heterozygosity of the *THRB* gene, encoding TRβ, was reported in human ECs [42]. Nevertheless, this loss of heterozygosity did not completely account for the reduction in TRβ1 expression [41]. Retinoid X receptor α (RXRα), known to form functional heterodimers with TRs, exhibits no change in expression levels between cancerous and normal esophageal mucosa [43]. Given that high selenium (Se) consumption has been shown to increase type I deiodinase activity, it is interesting that multivitamin/mineral (including 50 g/daily Se) intake was found to significantly decrease EC incidence in a nutritional intervention trial [44,45]. It remains unclear whether decreased risk of EC may be attributable to indirect effects of Se intake on TH signaling. Despite the reported correlations between various elements of thyroid hormone signaling and EC, studies have yet to prove direct causation.

In light of the apparent dichotomy between hyperthyroidism’s association with increased EC risk and the loss of TRβ1 in those cancers, further investigations are necessary to address the relationship between TH signaling and EC incidence. An increased susceptibility due to hyperthyroidism may be attributable to a receptor-specific mechanism. Thus, research regarding the therapeutic efficacy of specific receptor agonists or antagonists may prove beneficial. Future studies should also confirm whether selenium prevents EC, and if so, whether this occurs through alterations in TH processing or signaling.

## 3. Gastric Cancer

Several studies have investigated the role of TH in cancers of the stomach, although a precise relationship is yet to be established. Immunoblotting studies showed that alterations in TRα protein levels often occur in human gastric cancer (GC) and are associated with distant metastasis [46]. Significant differences were found in the incidence of autoimmune thyroid disease between subjects with GC and control subjects, although neither hyperthyroidism nor hypothyroidism was significantly associated with GC [47]. Correspondingly, another study demonstrated that a substantial number of patients with GC developed antithyroid antibodies, although this was not always associated with diminished thyroid function [48]. A higher incidence of atrophic body gastritis (a risk factor for GC) was observed in patients with autoimmune thyroid disease [49,50]. Likewise, administration of T_4_ to young, neonatally-thymectomized mice has been shown to reduce the incidence of autoimmune gastritis when administered during the active phase of the disease [51]. Further, as is the case with EC, intake of selenium-containing supplements is associated with a significant decrease in GC mortality, although it is not clear whether this is due to antioxidant properties of selenium or in part to effects on TH signaling [52]. Not all studies however, suggest a protective role for TH signaling. In one report, gastric cancer incidence in *N*-methyl-*N′*-nitro-*N*-nitrosoguanidine-treated Wistar rats was increased compared to untreated controls, with administration of T_4_ [53].

Thus, the specific effects of TH on GC incidence and underlying mechanism(s) require further examination. Differences in TH receptor levels may account for the contradictory results. Given that alterations in TRα are found in GCs, the effect of TRα-specific agonists such as CO23 on GC development and progression may be a fruitful target of future investigations [54,55]. Manipulation of particular TRs in gastric cancer cell lines, using specific targeting via siRNAs, also may provide insight into cellular TH response. As described above, nutritional interventions such as with Se could determine whether increased dietary supplementation with this trace element alone provides a protective effect against GC via changes in TH processing.

The association between goiter and the occurrence of GC is another focus area of investigation. Early analyses established iodine-deficient goiter as a risk factor for GC [56,57]. More recently a significant correlation between goiter and gastric noncardia adenocarcinoma was reported [58]. Another case-control study demonstrated that, in an area of Turkey with endemic iodine deficiency, patients with GC exhibit increased prevalence of goiter and autoimmune thyroid disease [47]. Accordingly, implementation of iodine prophylaxis has proven to be effective in decreasing the incidence rate and death rate for GC in iodine-deficient areas [59]. While mechanisms underlying these associations have not been identified, several hypotheses have been proposed. One hypothesis holds that iodine provides a protective effect against GC by acting as an antioxidant in the gastric mucosa [60]. Others have suggested, given the common embryonic origin of stomach and thyroid, that an iodine-deficient thyroid may produce a factor that causes chronic gastritis, leading to GC [61]. Thus, given the noted linkages between TH signaling, gastritis and GC, the effects of goiter-associated alterations in TH production remain a compelling question.

## 4. Hepatic Cancer

In contrast to the upper gastrointestinal tract, the impact of TH signaling on liver hepatocytes and hepatocellular carcinoma (HCC) has been well examined. T_3_ was found to stimulate hepatocyte proliferation and DNA synthesis in cell culture as well as in animal models of liver regeneration [62–64]. T_3_ stimulation in these cells led to downstream cyclin D1 induction [64,65]. In rats, experimental hyperthyroidism leads to increased hepatic expression of cyclins D1, E, and A as well as elevated Cyclin kinase (Cdk) activity and reduced Cdk inhibitor expression. Conversely, propylthiouracil (PTU)-induced hypothyroidism caused a decrease in cyclin D1 expression and Cdk activity in the same study [66]. Treatment of rats with T_3_ led to an induction of *VEGF* expression in liver cells (after partial hepatectomy), suggesting a role for T_3_ in reparative angiogenesis [61,63]. Since TRβ1 is the most abundant receptor isoform for TH in the liver, it is noteworthy that the TRβ-specific agonist GC-1 strongly stimulated cell proliferation in the rat liver [67,68]. Thus, the proliferative effect of TH signaling on hepatocytes is well documented.

Despite compelling evidence that T_3_ promotes mitosis in hepatocytes, this does not appear to be the case for hepatic cancer cells. Case-control studies demonstrate significant associations between hypothyroidism and HCC [69,70]. In livers of rats treated with diethylnitrosamine (DENA), T_3_ treatment reduced the number of glutathione *S-*transferase-positive hyperplastic lesions compared to controls, while also reducing the rate of HCC development and blocking metastases [71]. Similarly, administration of either T_3_ or the TRβ agonist GC-1 caused complete elimination of, or significant reduction in, numbers of pre-neoplastic liver lesions in DENA-treated rats [72]. In HepG2 sublines overexpressing either TRα1 or TRβ1, treatment with T_3_ reduced cell proliferation through activation of TGF-β, leading to down-regulation of Cdk2 and cyclin E and the up-regulation of p21 [73]. Interestingly, while T_3_ activated TGF-β to reduce HCC proliferation, it also promoted these cells’ invasive and metastatic potential. Treatment of HCC cells with T_3_ increased nuclear localization of Smad3/4, which then enhanced expression of furin, a proprotein convertase that processes latent precursor proteins into their biologically active products [74]. It is thought that T_3_ activation of TGF-β signaling may underlie enhanced tumor metastasis *in vitro* and *in vivo* [74]. In agreement with these findings, an early study showed that in PTU-induced hypothyroid rats, ^131^I administration, or surgical thyroidectomy significantly decreased both local and metastatic growth of Morris Hepatoma [75].

Substantial attention has been given to the roles of normal and mutated TH receptors in HCC development and progression. In human HCC specimens, a high prevalence of truncating mutations and point mutations are observed for both *TRα* and *TRβ* genes [76]. In human HCC cell lines, naturally-occurring mutations in *TRα1* and *TRβ1* resulted in reduced or absent transactivity [77]. Dominant negative *TRα1* mutants have also been isolated from tumors of HCC patients [78]. Many mutated TRs from HCC tumors retained their ability to repress TRE target genes in the absence of T_3_, but lacked T_3_-induced transactivation ability [79]. Several mutant TRs from HCC also failed to interact with their transcriptional corepressor proteins SMRT or NCoR, suggesting that association with these corepressors is not necessary for the dominant negative actions of TR mutants in these cancers [80]. It is possible that such mutants interfere with the anti-proliferative and anti-metastatic effects of wild type TRs in HCC cells. Indeed, mutated TRs in HepG2 cells showed significant alterations in their collections of target genes when compared to wild type counterparts [81]. The findings that mutated receptors contribute to HCC growth and progression in several models provide additional support for the case of TRs as tumor suppressors in HCC. Primarily, mutated *TRα* (v-ErbA) leads to the development of hypothyroidism and HCC in male mice [82]. Studies in cell lines and patient tumors have also implicated TH receptors in the induction of the tumor suppressor DKK4 as well as the repression of proto-oncogenes Sp1 and PTTG1 [83,84].

As the most abundant TR in hepatic tissue, TRβ has received particular attention for its role in HCC pathology. Expression of TRβ1 is highly correlated with increased invasive activity in human HCC cell lines and decreased expression of the anti-metastatic gene *nm23* [85]. However, a recent study showed that transfection of TRβ1 into human SK-hep1 cells reduced HCC xenograft tumor growth in nude mice, promoted partial mesenchymal-to-epithelial transition, attenuated tumor cell invasiveness, and blocked tumor cell responses to growth factors EGF, IGF-1, and TGF-β [86]. Interestingly, while growing tumors were found to lose TRβ1 expression, induction of hypothyroidism was associated with reduced tumor enlargement as well as an increased invasive and metastatic phenotype, regardless of whether the grafted cells expressed TRβ1 [87].

**Figure 1:**
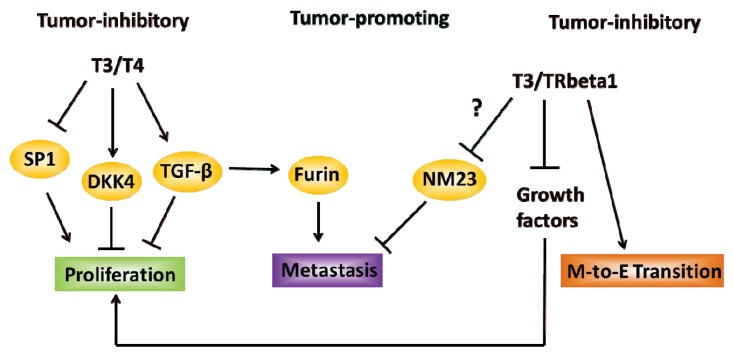
Current overview of the effects of TH signaling in liver cancers. T_3_/T_4_ was found to decrease cell proliferation by inducing the expression of tumor suppressors DKK4 and TGF-beta, while coincidentally suppressing the expression of proto-oncogenic SP1. Similarly, T_3_ bound to TRβ1 was observed to inhibit the proliferative effects of growth factors and to promote mesenchymal-to-epithelial (MET) transition. The promotion of TH signaling leading to invasion and metastasis may occur through TH-induced TGF-β activation of furin expression and of TRβ1-associated decreased expression of the anti-metastatic protein NM23.

Despite strong support for TH and its receptors in tumor suppression via Wnt inhibitor DKK4 induction and tumor oncogene Sp1 repression, the tumor-promoting effects of TH such as in increasing invasiveness via furin up-regulation and its negative association with expression of the gene *nm23*, thought to be a suppressor of metastasis, have also been reported (Figure 1) [74,83–85]. This presents a challenge to the overall understanding of TH signaling in hepatic cancer growth and progression, and needs to be resolved with further studies. It is tempting to speculate that such differences may be attributed to intra- or extra-cellular factors affecting the cellular response to T_3_. Moreover, the various roles of different TR isoforms under distinct physiological contexts might contribute to the noted discrepancies; a systematic evaluation of the efficacy of TR-specific agonists and antagonists, which may functionally recruit distinct co-regulators (co-activators or co-repressors) may help address this question.

## 5. Pancreatic Cancer

The putative relationship between TH signaling and pancreatic cancer (PaCa) has been the subject of several studies. A recent clinical study showed an association between hypothyroidism and autoimmune pancreatitis, the latter a potential risk factor for PaCa [88,89]. However, a cause-and-effect relationship has yet to be established. A similar study reported the significant occurrence of antithyroid autoantibodies in pancreatic adenocarcinoma patients, although circulating TH levels were not found to differ from controls. The latter raises the interesting possibility that the malignancy itself could be impairing immunoregulation, leading to the presence of autoantibodies [90]. In rats and mice treated with T_3_, increased proliferation of pancreatic acinar cells as measured by BrdU incorporation was demonstrated [91]. Similar to the effect seen in rat liver, the TRβ-specific agonist GC-1 induced cell proliferation in rat pancreatic cells *in vivo* [68]. Primary PaCa tumors with lymph node metastases exhibit significantly increased expression of integrin α_V_β_3_, a cell surface receptor for T_4_ [16,17,92]. Interestingly, tetrac, which inhibits binding of T_4_ to integrin α_V_β_3_, reduced PANC-1 tumor mass in a mouse xenograft model [18,19,22]. Tetrac also caused accumulation of the pro-apoptotic BcLx-s, reduction in tumor hemoglobin content (a marker for angiogenesis), and decreased expression of EGFR within the tumors [22].

Although T_3_ stimulates mitosis in normal pancreatic acinar cells, the association between TH concentrations or TR levels and pancreatic tumorigenesis remains unclear. Nevertheless, the tumor-suppressive and anti-angiogenic effects of tetrac are promising for PaCa treatment and prevention. Future investigations of tetrac’s effects on metastatic PaCa may be expanded into other mouse models, which may ultimately support the conduct of clinical trials. Given the preliminary efficacy of tetrac, prospective tumor-promoting functions of integrin α_V_β_3_ in PaCa also merit further exploration.

## 6. Colorectal Cancer

A number of association studies point to a preventive role of TH in the development of colorectal cancer (CRC). Hypothyroid patients who have used Levothyroxine for over five years are found to have significantly decreased relative risk of CRC [93]. Similarly, patients hospitalized for Graves’ disease, the most common form of hyperthyroidism, are at reduced risk for CRC development [94]. Further, plasma T_3_ levels are found to be reduced in CRC patients with systemic metastases, suggesting, though not proving, that TH signaling may suppress CRC invasiveness [95]. In addition to changes in circulating TH levels, changes in expression of TH receptors have been associated with CRC disease progression. In patient samples, colorectal tumors exhibit reduced expression of TRβ1 compared to matched normal mucosa. TRβ1 expression is associated with a more differentiated phenotype [96], consistent with the findings that loss of TRβ1 accompanies malignant transformation of human colon tumors [97]. Tumor vascular epithelial cell expression of integrin α_V_β_3_, the cell surface receptor for T_4_, is associated with reduced survival and increased metastatic potential in CRC [98]. Nevertheless, a protective role for TH has not been consistently supported in all studies. One study found a significant association between autoimmune thyroid disease and breast cancer, but not CRC [99]. This study also found no correlation between free T_4_ levels of serum and CRC, although a negative association was found within CRC patients between serum TSH levels and serum carcinoembryonic antigen, the latter a tumor marker [99]. Yet another group observed a significant association between high circulating TSH and cancer in general, but a lack thereof between TSH levels and CRC [100].

Effects of TH on intestinal cell and tumor cell proliferation and survival have been investigated in animal models. Male Sherman rats subjected to thyroidectomy exhibit fewer cells per crypt of Lieberkühn than do sham-operated control rats. Treatment of thyroidectomized rats with T_4_ in turn increased the number of cells per crypt, confirming a proliferative role for T_4_ in the intestinal stem-progenitor cell compartment [101]. Binding of T_3_ to TRα1 directly stimulates expression of β-catenin, a major driver of intestinal cell proliferation [102,103], which occurs through TRα1 activation of the Frizzled-related protein sFRP2 [104]. Further, mice null for TRα display a sustained apoptotic response to DNA damage consequent to a delay in p53 phosphorylation, suggesting that TRα plays a critical role in regulating intestinal cell renewal and apoptosis [36]. Indeed, mice with intestinal epithelium-targeted overexpression of TRα1 (*vil-*TRα1) exhibit aberrant architecture of the intestinal mucosa as well as significantly increased cell proliferation. While these mice do not manifest spontaneous intestinal cancers, *vil-*TRα1/Apc^+/1638N^ mice develop tumors at a higher rate than Apc^+/1638N^ mice, supporting the participation of TRα1 in the promotion of intestinal tumorigenesis [105]. In another model of colon tumorigenesis, rats treated with azoxymethane (AOM) along with T_4_ showed an increased incidence of colon tumors than those treated with AOM alone [106]. In addition to the actions of canonical TH receptors, treatment of mice with the integrin α_V_β_3_ antagonist S247 reduced liver metastasis in a colon cancer xenograft model [107].

The role of the iodothyronine deiodinases in CRC development and progression has also been investigated. Type III iodothyronine deiodinase (D3) is of particular interest. A number of cancer cell lines including endometrial carcinoma, mammary carcinoma, neuroblastoma, and CRC express elevated D3 levels [108,109]. Expression of D3 is increased in human colon adenomas and adenocarcinomas compared to healthy surrounding mucosa [110]. In CRC cell lines, the β-catenin/TCF complex transcriptionally induces D3. Knockdown of β-catenin reduced D3 expression levels and concomitantly induced expression of D2. Under these conditions, T_3_ reduced proliferation and enhanced differentiation of CRC cells in both cell culture and xenografted mice [110].

As is the case for other cancers of the digestive tract, there is compelling evidence that Se intake reduces the risk of developing CRC. In a randomized placebo-controlled trial, Se supplementation reduced CRC incidence by 58 percent, relative to controls [111]. Other studies showed an association of blood Se levels with decreased CRC incidence [112,113]. In this regard, a study of Se intake in mice suggested that colon D1 expression/activity is unchanged with Se supplementation, whereas D2 and D3 transcripts are undetectable in the colon when measured by either microarray or qPCR [114]. Thus, it is possible that mucosal protein expression/activity of D3 is elevated by Se, to antagonize T_3_ anti-proliferative effects. In support of this, Se deficiency is associated with increased colon expression of β-catenin, Dvl2, and c-Myc as well as decreased expression of Gsk3β [114]. Thus, increased β-catenin signaling may lead to up-regulation of D3 to decrease intracellular T_3_ levels and hence, its growth-inhibitory effects.

**Figure 2:**
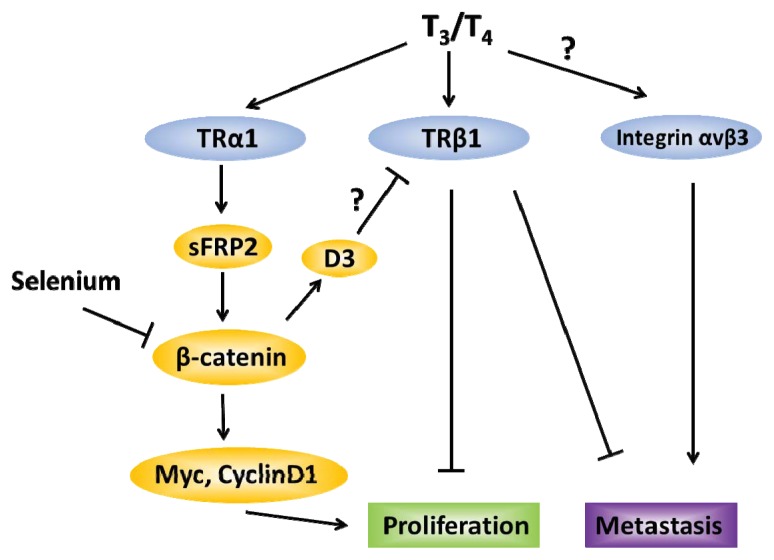
Schematic representation of downstream effects due to T_3_/T_4_ binding to distinct receptors in colorectal cancer. When bound to TRα1, TH induces sFRP2, in turn activating β-catenin and cellular proliferation. Colon β-catenin expression is reduced by selenium intake, and can induce expression of D3, which then antagonizes the anti-proliferative effects of T_3_ (possibly through interactions with TRβ1). TRβ1 is associated with cellular differentiation and reduced metastasis. The TH surface receptor integrin αvβ3, on the other hand, is associated with reduced CRC survival and increased metastasis.

Figure 2 summarizes the opposing effects of TRβ1 (tumor-suppressive) and TRα1 (tumor-promoting) in CRC. The systematic evaluation of the efficacy of TRβ1-specific agonists such as GC-1 and KB2115 in colon cancer prevention and treatment is noteworthy, particularly in human clinical trials [115,116]. Given that body mass index is positively correlated with incidence of various cancers, the noted anti-obesogenic effects of these agonists may contribute to cancer prevention [115–117]. Similarly, TRα1-specific antagonists such as debutyldronedarone as well as the anti-metastatic integrin α_V_β_3_ antagonist S247 are ripe for further examination as potential inhibitors of colon cancer metastasis [107,118]. Finally, given that D3 and other deiodinases as well as Se intake may impact TH signaling, their relevance to CRC prevention and treatment are fruitful avenues to pursue with the goal of improving CRC outcome.

## 7. Concluding Remarks and Future Directions

Evidence has implicated components of TH signaling in the development and progression, as well as in the prevention of various cancers of the alimentary tract. While some cancers such as HCC and CRC have been the subjects of numerous TH-related investigations, the roles of TH in other cancers such as PaCa and EC are less established. Thus, there are many future research avenues to pursue in order to address the gaps of knowledge in this field. One such avenue pertains to the relative absence of knowledge of functions and downstream effects of each TR and their agonists/antagonists in the digestive system and the clinical feasibility of using these molecules, taking into consideration potential off-target effects. There is a large ongoing effort geared towards developing next generation TR-specific agonists and antagonists for use in obesity and fatty liver disease therapies; such molecules also may have applicability in gastrointestinal cancer prevention and treatment. A second direction of research concerns the putative role of TH signaling in the normal biology and pathobiology of intestinal stem and progenitor cells, potential contributors to oncogenic transformation [119–121]. This also is a possible biological context by which TR mutations aid in cancer initiation and progression. A third important area of investigation is to provide confirmation of the relevance of integrin α_V_β_3_ in gastrointestinal neoplasia, given its emerging role as a mitogenic cell surface receptor for TH. Fourth, in the case of HCC, studies should seek to identify the mechanistic rationale and biological and physiological contexts for the contrasting tumor-promoting and tumor-inhibitory actions of TH (Figure 1). Research to assess the functions of iodothyronine deiodinases in cancers and molecular mechanisms underlying the presumed preventive effects of micronutrients (Se, iodine), as well as novel natural products and bioactive dietary factors with pro- and anti-TH activities, may eventually lead to targeted therapeutic strategies against digestive tract cancers [122]. Lastly, we know very little about TR co-regulators and their functions in the digestive system, and whether these molecules (or their absence) play a role in cancer development.
